# Evaluation of Metabolomic Changes as a Biomarker of Chondrogenic Differentiation in 3D-cultured Human Mesenchymal Stem Cells Using Proton (^1^H) Nuclear Magnetic Resonance Spectroscopy

**DOI:** 10.1371/journal.pone.0078325

**Published:** 2013-10-21

**Authors:** Moo-Young Jang, Song-I Chun, Chi-Woong Mun, Kwan Soo Hong, Jung-Woog Shin

**Affiliations:** 1 Department of Biomedical Engineering/UHRC, Inje University, Gimhae, Gyeongnam, South Korea; 2 Graduate School of Health Science and Technology, Inje University, Gimhae, Gyeongnam, South Korea; 3 Korea Basic Science Institute, Ochang, Chungbuk, South Korea; Northwestern University, United States of America

## Abstract

**Purpose:**

The purpose of this study was to evaluate the metabolomic changes in 3D-cultured human mesenchymal stem cells (hMSCs) in alginate beads, so as to identify biomarkers during chondrogenesis using ^1^H nuclear magnetic resonance (NMR) spectroscopy.

**Materials and Methods:**

hMSCs (2×10^6^ cells/mL) were seeded into alginate beads, and chondrogenesis was allowed to progress for 15 days. NMR spectra of the chondrogenic hMSCs were obtained at 4, 7, 11, and 15 days using a 14.1-T (600-MHz) NMR with the water suppression sequence, zgpr. Real-Time polymerase chain reaction (PCR) was performed to confirm that that the hMSCs differentiated into chondrocytes and to analyze the metabolomic changes indicated by the NMR spectra.

**Results:**

During chondrogenesis, changes were detected in several metabolomes as hMSC chondrogenesis biomarkers, e.g., fatty acids, alanine, glutamate, and phosphocholine. The metabolomic changes were compared with the Real-Time PCR results, and significant differences were determined using statistical analysis. We found that changes in metabolomes were closely related to biological reactions that occurred during the chondrogenesis of hMSCs.

**Conclusions:**

In this study, we confirm that metabolomic changes detected by ^1^H-NMR spectroscopy during chondrogenic differentiation of 3D-cultured hMSCs in alginate beads can be considered as biomarkers of stem cell differentiation.

## Introduction

Regenerative medicine is a subdiscipline of tissue engineering, which aims to restore or establish normal functions of injured tissues and organs using human genes, proteins, and cells. This approach may involve the regeneration of tissues and organs by injecting stem cells or progenitor cells (cell therapies) and/or transplantation of *in vitro*-grown tissues [1]. Stem cells are able to divide and differentiate into different cell types. In adult organisms, stem cells have the ability to repair damaged tissues by regenerating autologous cells [2-4]. However, it is well recognized that some tissues or organs, such as adult articular cartilage, have a limited capacity for self-repair [5,6]. A previous study reported that the use of autologous chondrocyte transplantation (ACT) and cell-based therapy for healing articular cartilage defects minimized potential side-effects and was successfully employed in the clinical setting [6-8].

The identification of surrogate markers and biomarkers to provide structural, physiologic, and metabolomic information regarding cell status is essential for the assessment of therapeutic interventions in regenerative medicine [9]. Stem cell behavior cannot be easily addressed *in vivo* because exogenous cell markers are lost through degradation, dilution, and excretion as cell populations divide [10]. Accordingly, research into novel noninvasive biomarkers or surrogate parameters to visualize or analyze quantitatively them *in vivo* is expected to provide innovative diagnostic information without causing stress or physical trauma to patients. These new techniques are also expected to facilitate investigations of cellular metabolism without causing detrimental effects [9,11,12]. Early detection of biomarkers of treatment efficacy at the cellular or molecular level is crucial and challenging in clinical applications of regenerative medicine [13].

Microscopic anatomic information about cells and tissues is usually obtained through histologic analyses. However, most of these techniques require an invasive process, such as extraction of tissue, formalin fixation, and chemical reactions that cause denaturalization by reaction with reagents [14-18]. Nuclear magnetic resonance spectroscopy (NMR), which is another powerful tool for molecular structural characterization, can be used to acquire metabolomic information from tissues [19,20]. Several investigators have attempted to establish a noninvasive biomarker by observing cell activities, such as cell death or differentiation, using NMR [13,20,21]. In clinical applications, magnetic resonance spectroscopy (MRS) offers the potential to identify such noninvasive biomarkers by the detection of *in vivo* metabolic changes occurring in abnormal tissues prior to the alterations becoming apparent by magnetic resonance imaging (MRI). However, clinical MRS analyzes only a low number of metabolomes, and the cellular mechanisms underlying alterations are poorly understood. *In vitro* MRS or NMR studies allow systematic assessment of many metabolomes, which may provide valuable information about cellular processes [13].

In the present study, metabolomic changes in three-dimensional (3D)-cultured human MSCs (hMSCs) immobilized in alginate beads were evaluated during chondrogenic differentiation to identify biomarkers using ^1^H-NMR.

## Materials and Methods

### Cell culture

Bone marrow-derived hMSCs (Cambrex BioScience Co., Walkersville, MD, USA) were cultured in Mesencymal Stem Cell Growth Medium (MSCGM) that contained L-glutamine, gentamicin sulfate, amphotericin B, and mesenchymal cell growth supplement (Lonza Ltd., Portsmouth, NH, USA) and incubated at 37°C in an atmosphere of 95% air and 5% CO_2_. The medium was replaced every 4–5 days. Subconfluent cells were subcultured using trypsin (Lonza Ltd.) for six passages [22].

### 3D chondrogenesis culture

Trypsinized hMSCs were centrifuged at 1500 rpm for 3 min. The cells were resuspended in chondrogenic induction medium (CIM; Dulbecco’s Modified Eagle’s Medium [DMEM] high glucose, ITS+ [Becton Dickinson Co., Franklin Lakes, NJ, USA] at 100:1 dilution, ascorbic acid at 50 µg/mL [Sigma Chemical Co., St. Louis, MO, USA], transforming growth factor-β3 at 10 ng/mL [Peprotech Inc., Rocky Hill, NJ, USA], and dexamethasone at 100 nM [Sigma]) [22-24]. A suspension of 2×10^6^ cells was mixed with an equal volume of 2.4% (w/v) alginate gel. Alginate beads were formed using a syringe with a 23-G needle and solidified using 102 mM CaCl_2_ for 5 min. The alginate beads were washed three times with phosphate-buffered saline (PBS). The final cell density in the alginate beads was 2×10^6^ cells/mL. 

### Alginate bead lysis

Alginate beads were lysed to collect cell pellets for Real-Time PCR and cell counting after the acquisition of NMR data 20 alginate beads per experimental group were dissolved in lysis solution that contained 150 mM sodium chloride and 55 mM sodium citrate. The mixtures were incubated for 5–10 min at 37°C in 5% CO_2_ [25]. The alginate beads were released by pipetting and were centrifuged. After the supernatant was aspirated, the pellet was suspended in PBS, and the number of cells therein was counted.

### NMR sensitivity test sample preparation

The optimal cell density for MRI signal acquisition was investigated by scanning alginate beads that contained the following numbers of hMSCs per mL: 5×10^4^, 5×10^5^ (low), 2×10^6^, 5×10^6^ (intermediate), and 1×10^7^ (high). In general, ~10^5^ cells/mL was considered to be a low density of cells, ~10^6^ cells/mL an intermediate density, and ~10^7^ cells/mL a high density [7,26,27]. Three samples per group were prepared in 5-mm NMR tubes (Optima Inc., Elk Grove Village, IL, USA).

### NMR sample preparation

D_2_O saline (0.9% NaCl in 99.9% deuterium water, D_2_O) was used as the NMR locking solvent to maintain the osmotic pressure of the cells. Cultured alginate beads were washed in D_2_O saline three times before NMR scanning, to remove the water signal from the medium. Washed alginate beads were placed in 5-mm NMR tubes with D_2_O saline that contained 1 mM TSP (3-[trimethylsilyl] propionic-2,2,3,3-d_4_ acid sodium salt) in a 500-μL volume. TSP was used as a NMR standard reference material for calibration of the 0-ppm spectral peak position.

### ^1^H-NMR data acquisition and processing

NMR data were acquired using a 600-MHz micro-imaging/NMR machine (14.1 T, DMX 600; Bruker Co., Karlsruhe, Germany). Spectra were obtained using the water suppression sequence, zgpr (Bruker standard sequence). The 90° pulse calibration and shimming were conducted based on each sample before data acquisition. A repetition time of 2 s and data acquisition point of 16000 were used. The number of scans was 512 for the acquisition of each spectrum. The final scan time was 24 min, 51 s. The acquired data were processed using NUTS (Acorn NMR Inc., Livermore, CA, USA). A line broadening of 2 Hz was applied to the FID data before Fourier transform (FT). Baseline and phase correction were performed after FT. All spectra were normalized to the TSP peak to quantify the metabolomic changes that occurred during the differentiation of hMSCs.

### RNA purification and Real-Time PCR

RNA was prepared from hMSCs and chondrogenic hMSC pellets that had been washed three times with PBS. RNA was purified using an RNA purification kit (RNeasy Mini kit; Qiagen, Hilden, Germany) and converted to cDNA using a high-capacity RNA to cDNA kit (Applied Biosystems Inc., Foster, CA, USA). Real-Time PCR was performed using primers designed with the Primer Express 3.0 software (Applied Biosystems). The mRNAs that encode GAPDH, collagen type I, and collagen type II were amplified. Table 1 lists the primer sequences. Real-Time PCR for the measurement of gene expression levels was performed using forward and reverse primers in a SYBR green PCR master mix (Applied Biosystems), as described previously [24,28].

**Table 1:** Description of primers designed for PCR.

**Organism**	**Gene**	**Accession No.**	**Primer sequence, 5′3′**
	Collagen type I	NM_000088.3	Forward: CAGACAAGCAACCCAAACTGAA
			Reverse: TGAGAGATGAATGCAAAGGAAAAA
*Homo*	Collagen type II	NM_001844.4	Forward: GCCAACGTCCAGATGACCTT
*sapiens*			Reverse: CTTGCAGTGGTAGGTGATGTTCT
	GAPDH	NM_002046.3	Forward: CCAGGTGGTCTCCTCTGACTTC
			Reverse: GTGGTCGTTGAGGGCAATG

### DNA assays and cell counting

DNA assays and cell counting were performed to verify the optimal number of cells required for NMR analysis, which was determined using the NMR sensitivity test. Cell pellets for DNA assays were lysed using 0.1% Triton X-100 and centrifuged at 13,000 rpm at 4°C. Then, 100 μL of the supernatant were transferred to the wells of a 96-well plate with 100 μL of PicoGreen fluorescence reagent (Invitrogen Co., Grand Island, NY, USA). DNA standards were prepared using the same procedure. Fluorescence was measured in a multi-detection reader and was compared with the standard DNA curve. Cells extracted from alginate beads were dissolved in 1 mL PBS and enumerated using a hemocytometer. The intensities of NMR spectra from chondrogenic hMSCs cultured in alginate beads were normalized to cell density.

### Statistical analysis

All NMR experiments were performed in duplicate. Thus, all the results were derived from two independent experiments, each of which included at least three samples. One-way ANOVA tests were used to analyze multiple sets of data at a significance level of *p* < 0.05 with Leven’s homogeneity test and *post-hoc* Tukey’s or, where appropriate, Dunnett’s T3 test [29]. Statistical analysis was conducted using the SPSS ver. 16.0 software (IBM Co., Armonk, NY, USA). Furthermore, we investigated the relationship between chondrogenesis and metabolomic changes using the NMR spectra obtained from repeated experiments, to identify a biomarker of hMSC chondrogenesis.

## Results

### NMR sensitivity

**Figure 1:**
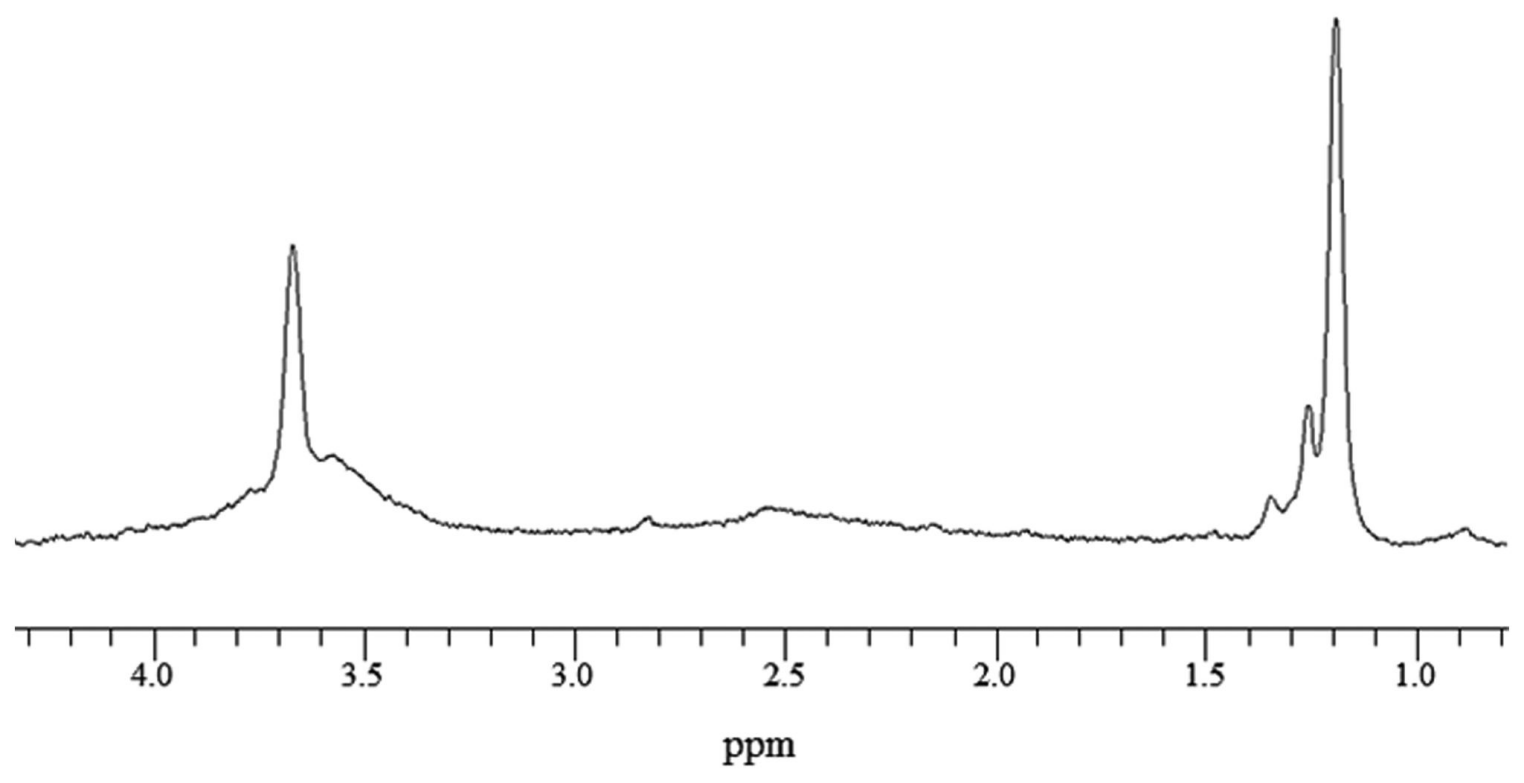
NMR spectrum of alginate beads without cells.

**Figure 2:**
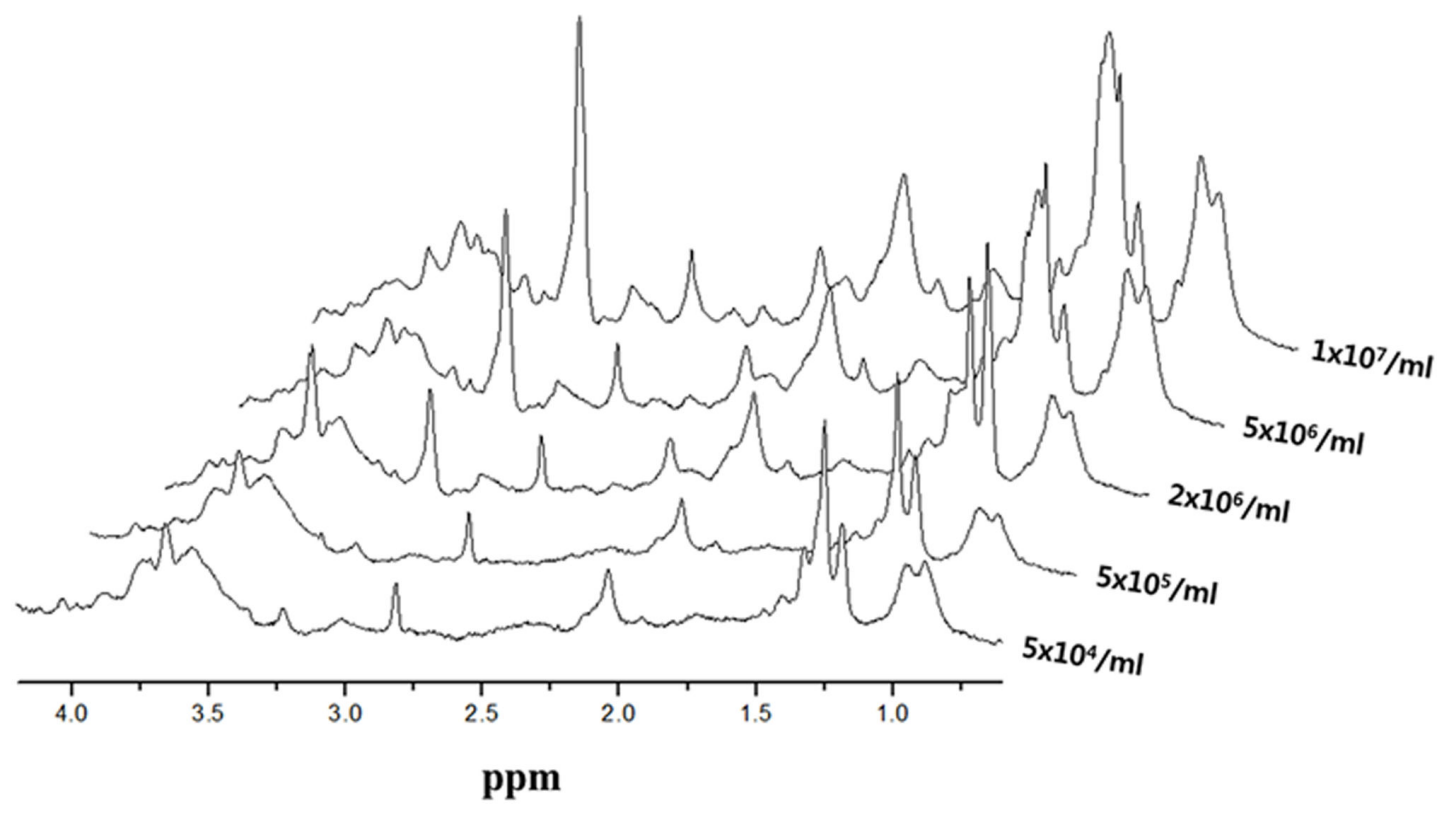
NMR spectrum changes of hMSCs according to cell density.

The NMR spectrum of alginate without cells was acquired as a reference (Figure 1). Linear intensity changes on NMR spectra according to cell density were observed by comparing the integral value of cell metabolomic peaks based on 1 mM TSP. Cell metabolomes, glutamate at 2.35 ppm, and phosphocholine at 3.22 ppm (Figure 2) were observable with the naked eye when the cell density was ≥ 2×10^6^ cells/mL. Table 2 shows the quantitative analysis of each metabolome. Increment ratios (%) of peak intensity were compared based on samples with 5×10^4^ cells/mL. Increments of spectral peak areas > 20% were confirmed using samples with 2×10^6^ cells/mL.

**Table 2:** Ratios of metabolite peak changes based on 5×10^4^ cells/mL (%).

**ppm**	**5×10^5^ cells/mL**	**2×10^6^ cells/mL**	**5×10^6^ cells/mL**	**1×10^7^ cells/mL**
3.22	5.7	44.3	63.7	77.2
3.01	0.8	19.4	33.5	53.1
2.35	13.6	36.9	50.2	57.1
2.04	14.11	31.2	49.9	63.9
1.70	2.3	23.6	49.2	54.5
1.48	2.0	35.1	41.1	56.2
1.30	4.2	50.1	65.0	78.6

### Normalization of NMR spectra to cell density

**Figure 3:**
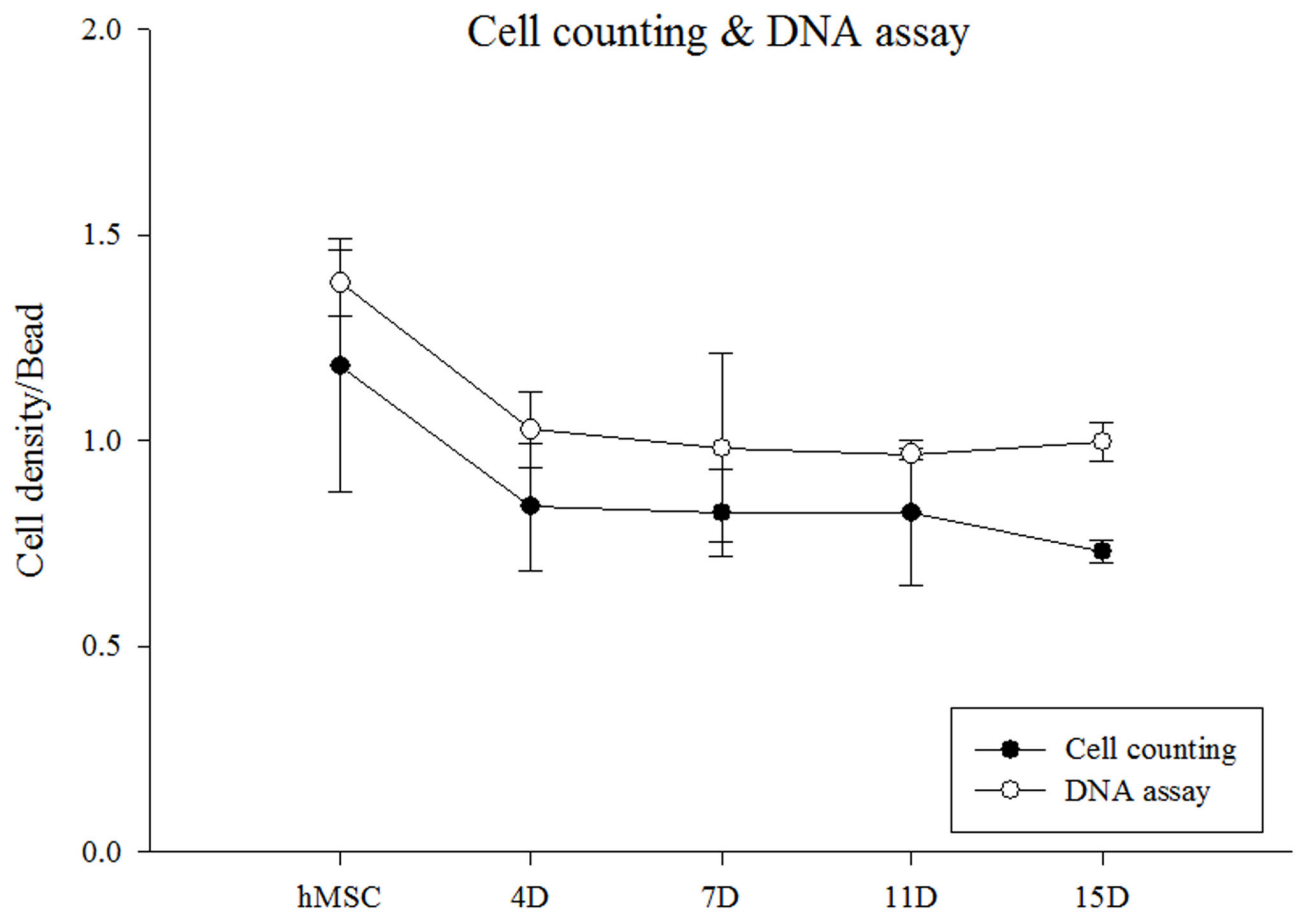
Variation of cell density per alginate bead during chondrogenesis.

Cell counting and DNA assays were performed to measure the variations in cell density during chondrogenesis. Both methods gave similar trends, as shown in Figure 3. The hMSC density varied during the 15 days of differentiation into chondrocytes. Metabolomic peak intensity changed linearly with cell density during chondrogenesis. All the spectra were normalized to the cell counts.

### Spectral analysis

**Figure 4:**
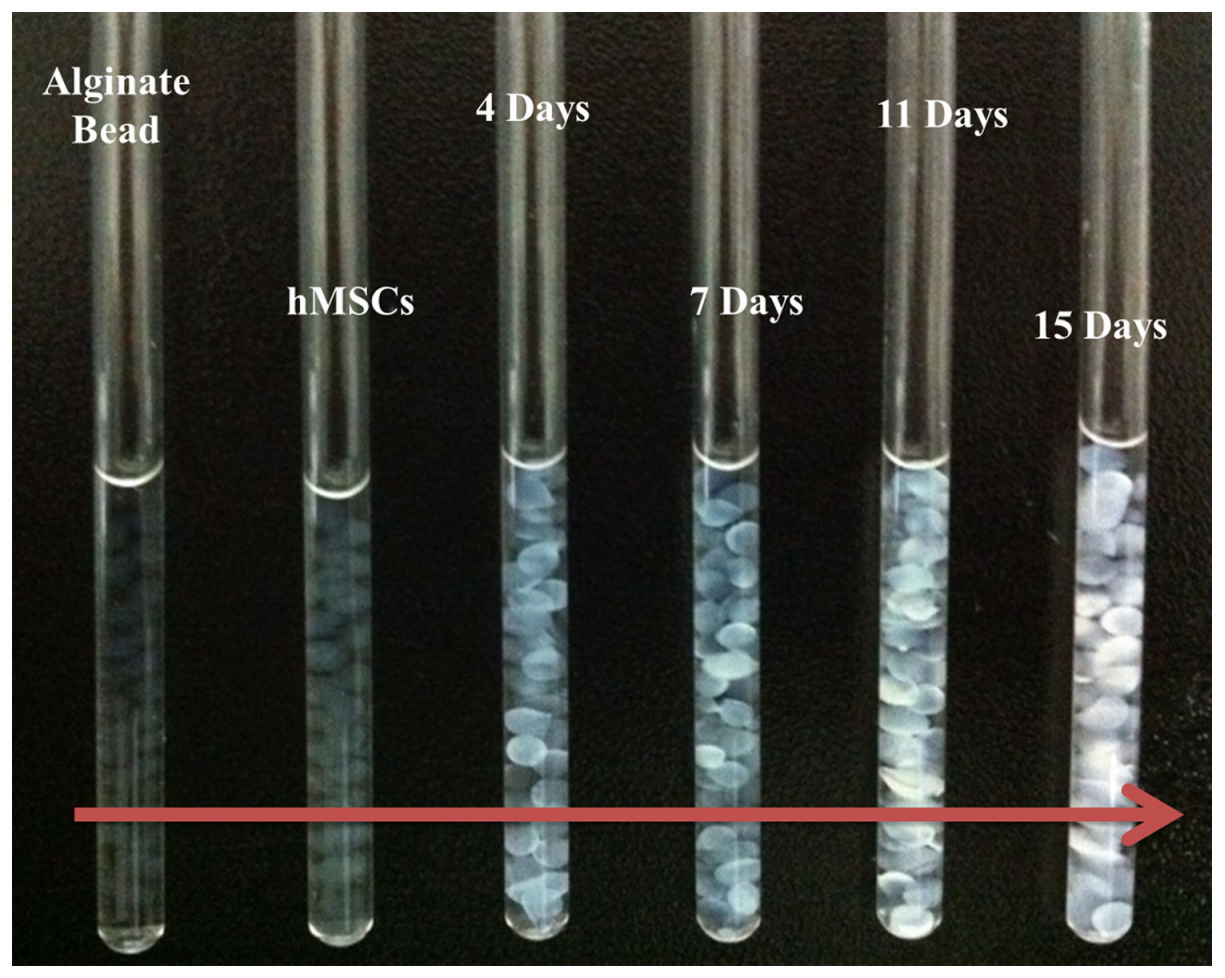
Chondrogenic hMSCs in alginate beads in an NMR tube.

**Figure 5:**
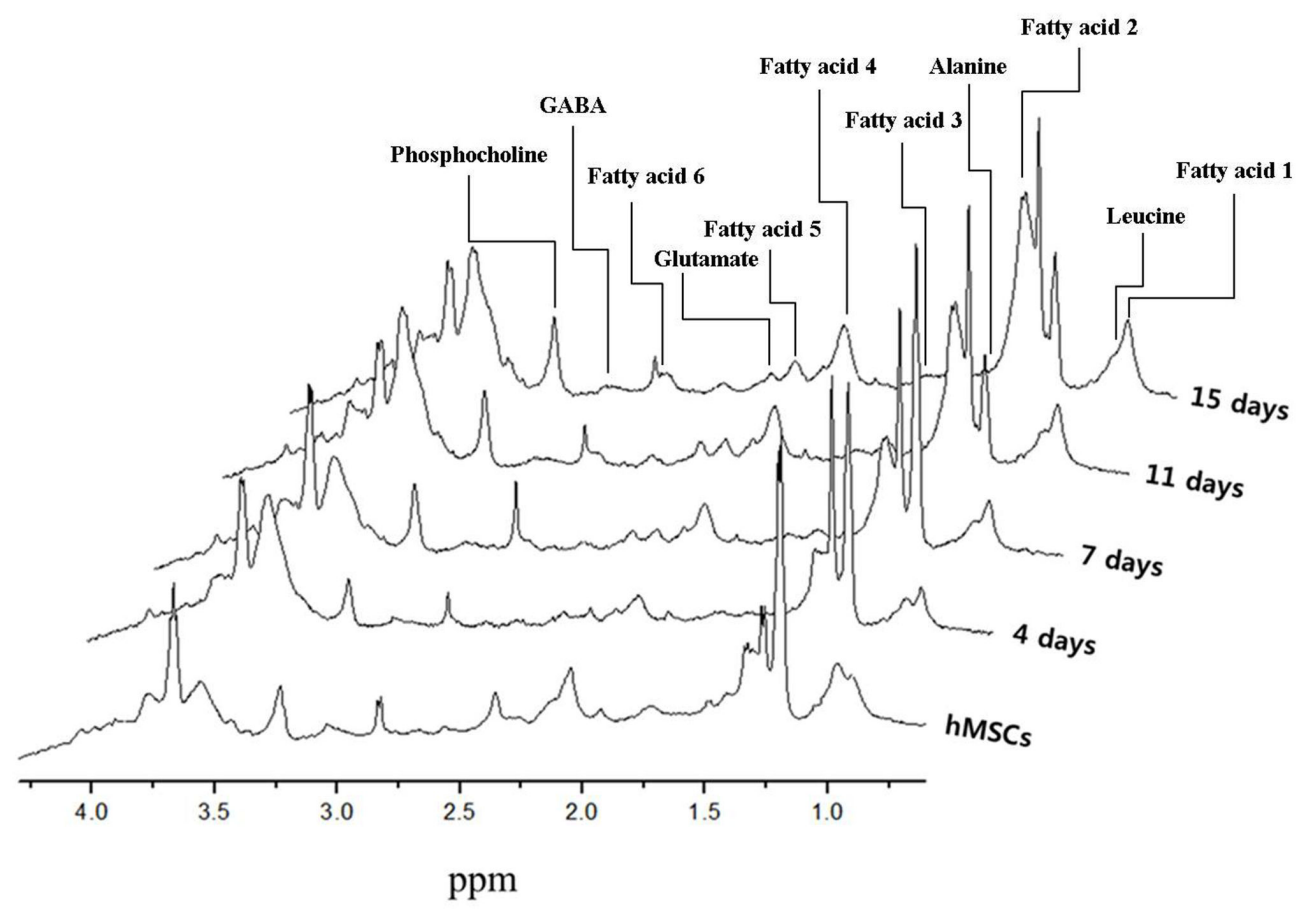
3D perspective of NMR spectra of chondrogenic hMSCs cultured on alginate beads.

Figure 4 shows a photograph of chondrogenic hMSCs on alginate beads in an NMR tube used for acquiring NMR spectra. The alginate beads changed from a deep white color to appearing transparent during hMSC chondrogenesis. Three-dimensional perspective plots of the NMR spectra acquired at 4, 7, 11, and 15 days are shown in Figure 5.

**Figure 6:**
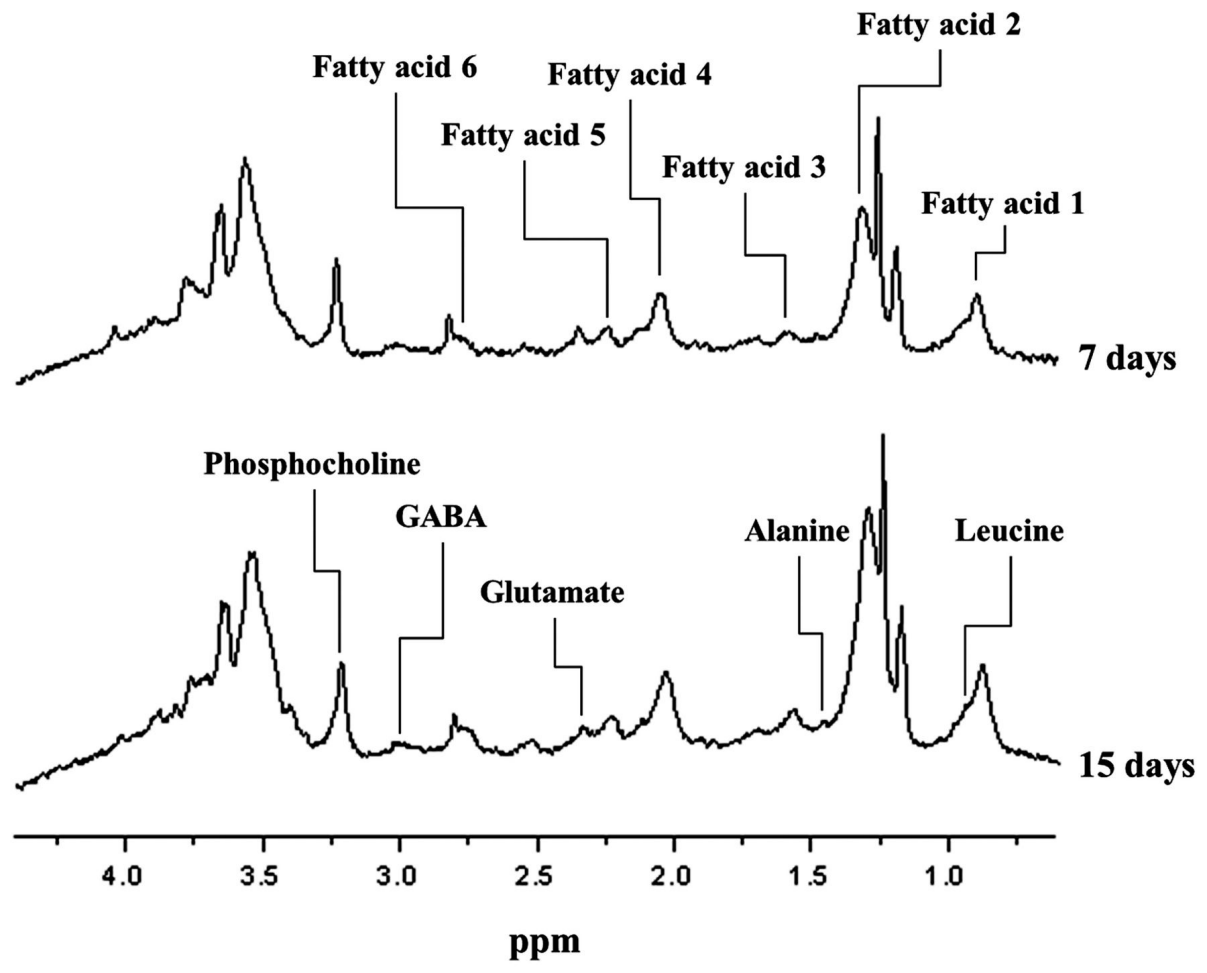
Displayed metabolomics on NMR spectra of chondrogenic hMSCs.

All metabolomic peaks derived from the chondrogenic hMSCs were selected, with the exception of the alginate peak shown in Figure 1. Cell metabolomic changes during chondrogenic differentiation of hMSCs were quantitatively analyzed by comparing the integral values of the peaks of the normalized NMR spectra. The metabolomes of the following changed during chondrogenesis: six fatty acids (see Table 3 and upper spectrum in Figure 6); leucine; alanine; glutamate; GABA (γ-aminobutyric acid); phosphocholine; and creatine (see Table 4 and lower spectrum in Figure 6) [20,21,30-32]. 

**Table 3:** Fatty acids changed during hMSC chondrogenic differentiation.

**Fatty acid**	**ppm**	**Chemical structure**
1	0.85–0.92	-CH_3_
2	1.26–1.32	-(CH2)_n_-
3	1.54–1.63	-OOC-CH_2_-CH_2_- + -O-CH_2_-CH_2_- + -CH_2_-CH-(CH_3_)_2_-
4	1.99–2.10	=CH-CH_2_-CH_2_ + =CH-CH_2_-CH_3_
5	2.20–2.26	-COO-CH_2_-CH_2_-
6	2.81–2.82	-CH=CH_2_-CH=

**Table 4:** Changes in levels of non-fatty acid metabolites during chondrogenesis.

**Name**	**ppm**	**Chemical structure**
Leucine	0.95	δCH_3_
	0.96	δ'CH_3_
Alanine	1.48	βCH_3_
Glutamate	2.35	γCH_2_
GABA	3.01	2CH_2_
Creatine	3.03	CH_3_
Phosphocholine	3.22	N-(CH_3_)_3_

**Figure 7:**
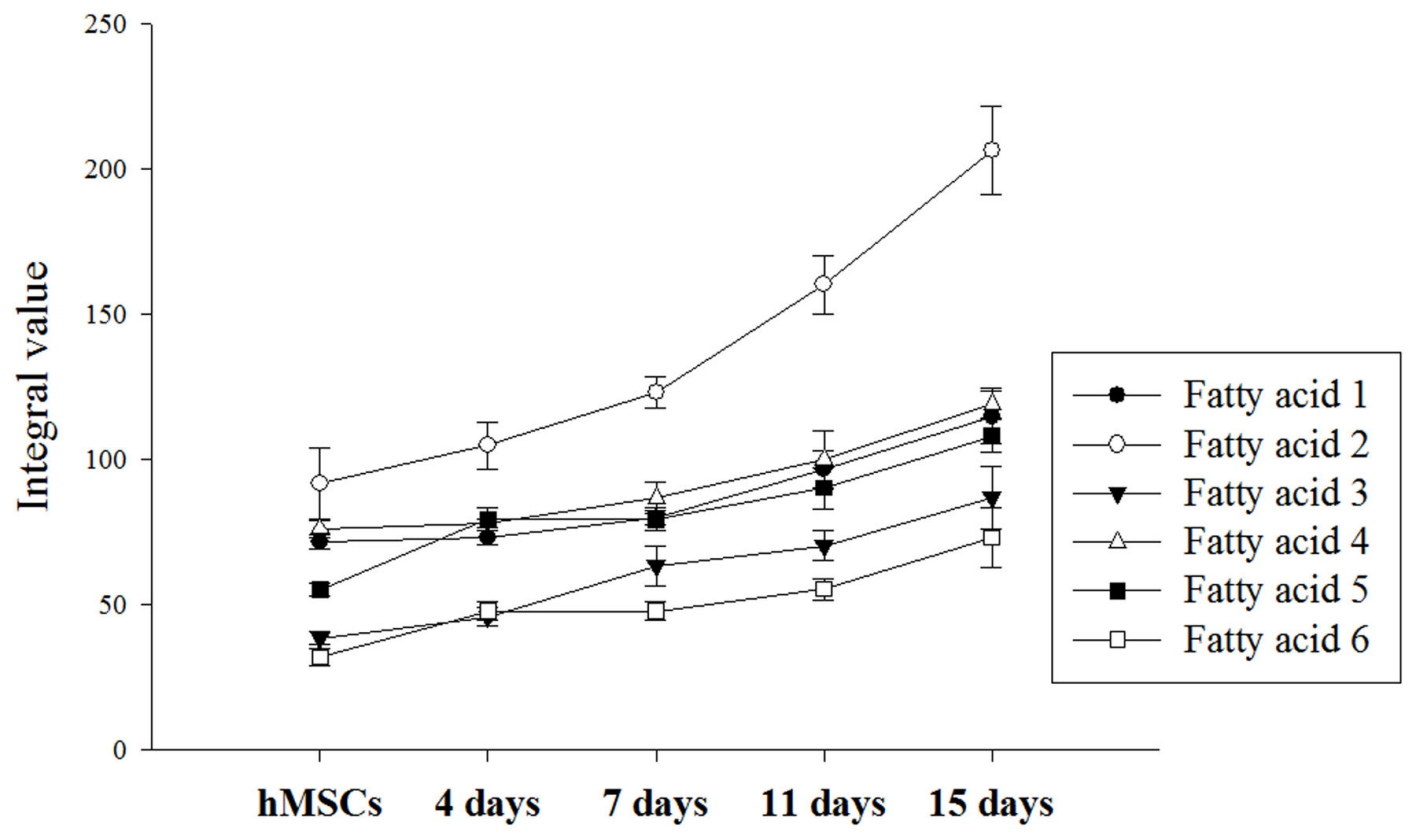
Fatty acid changes during chondrogenesis.

Figure 7 shows that levels of all of the fatty acids increased during chondrogenic differentiation. Fatty acid 3 at 1.58 ppm and fatty acid 6 at 2.75 ppm had the lowest initial values, but they increased rapidly starting on Day 4. In addition, both peaks were not evident the naked eye in the hMSC NMR spectrum, although it was confirmed that these peaks gradually increased after the start of chondrogenesis. The most significantly increased metabolome was fatty acid 2 at 1.30 ppm. This fatty acid doubled its starting concentration during the 15 days of chondrogenic differentiation. The chemical structure of each fatty acid is described in Table 3.

**Figure 8:**
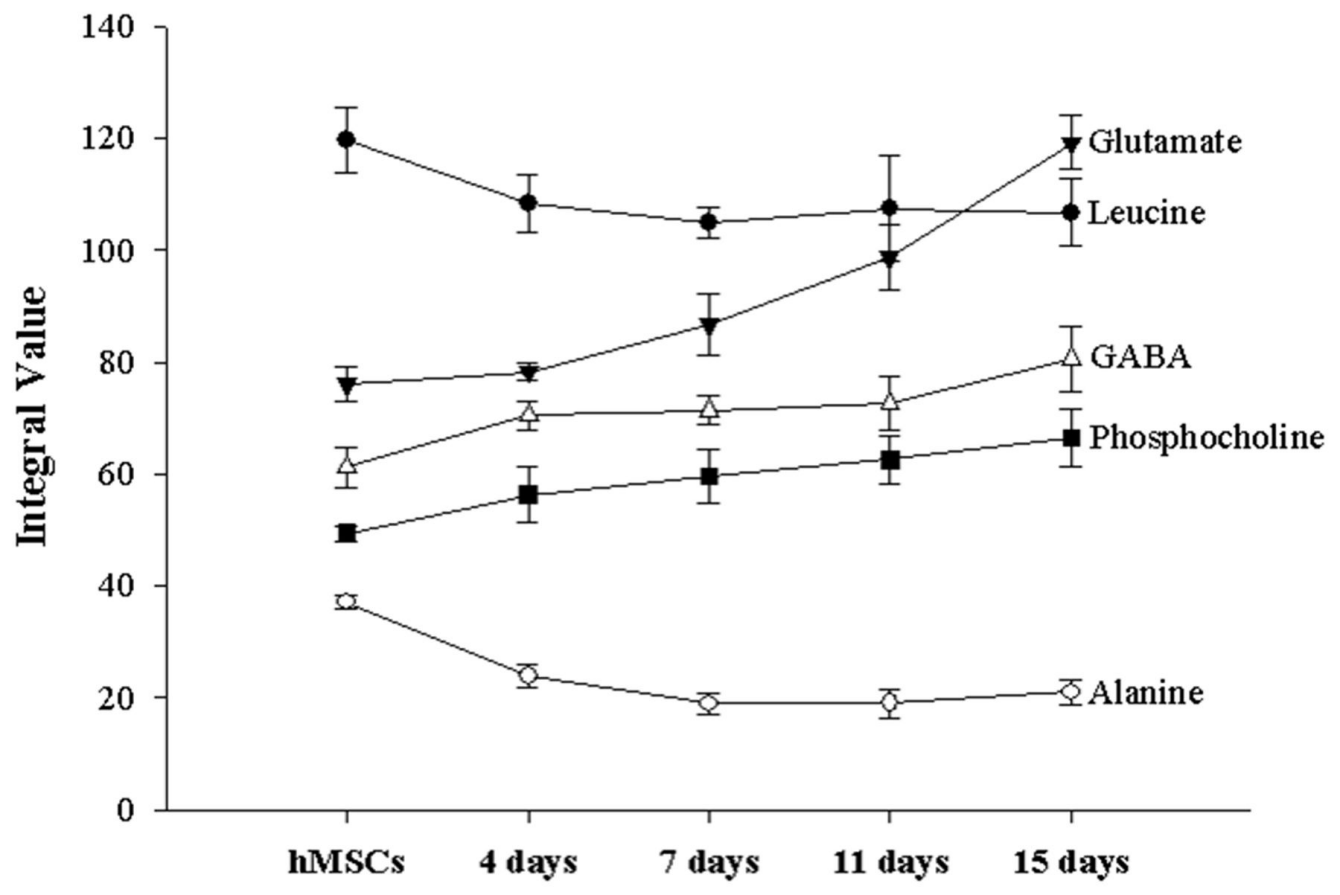
Changes in levels of non-fatty acid metabolites during chondrogenesis.

Other metabolomic changes were also observed (Table 4 and Figure 8). The metabolomes of leucine and alanine decreased 4-7 days after the initiation of chondrogenesis. In contrast, the levels of GABA, phosphocholine, and glutamate increased during chondrogenic differentiation. Creatine was detected only in pre-differentiated hMSCs.

### Statistical analysis

**Figure 9:**
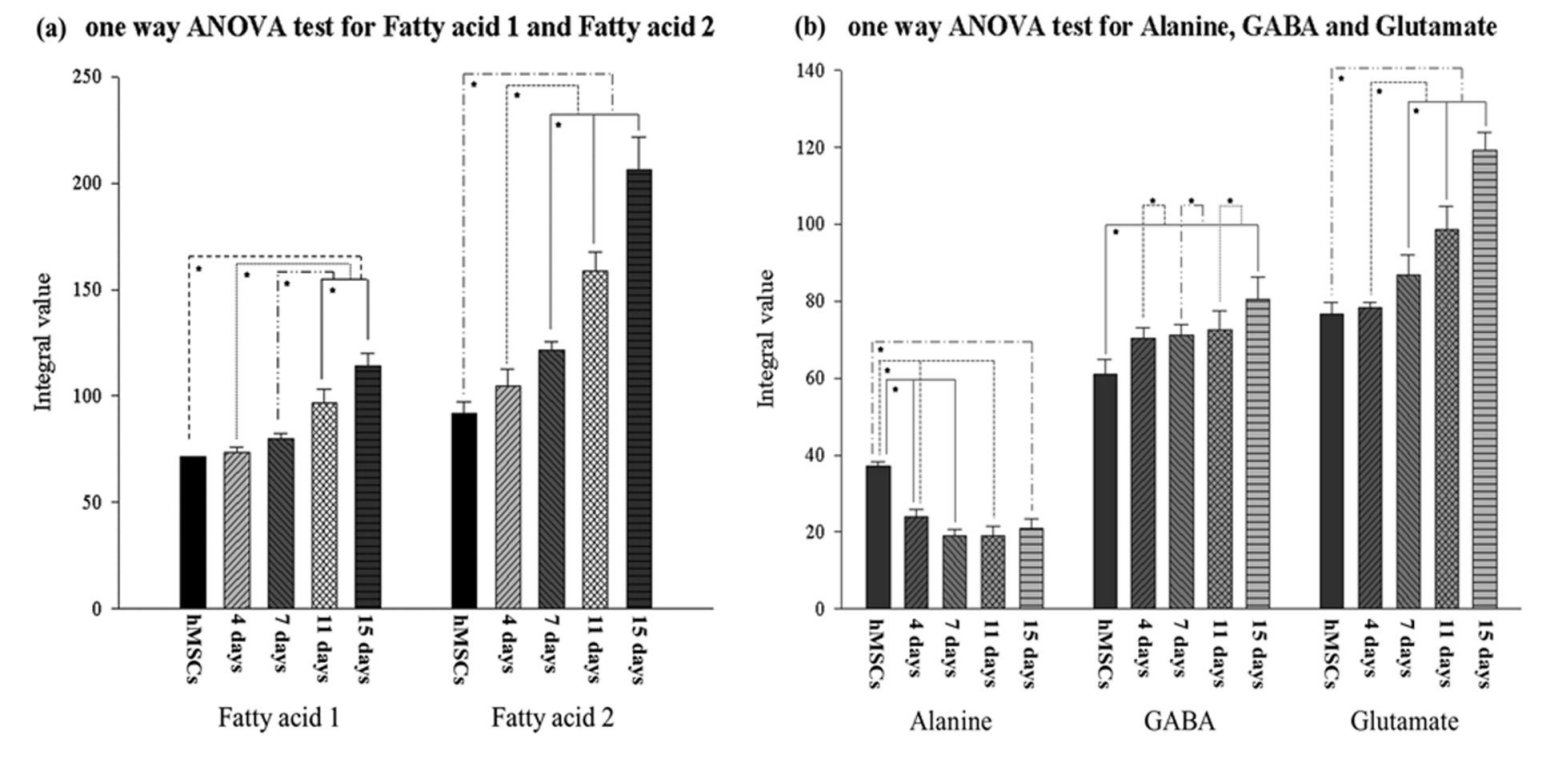
One-way ANOVA of cell metabolites according to duration of chondrogenic differentiation. (a) Fatty acid 1, Tukey’s *posteriori* test; fatty acid 2, Dunnett’s T3 *posteriori* test. Statistical tests were performed for both the first and second hMSC chondrogenic differentiation experiments. (b) Alanine, GABA and glutamate. Tukey’s *posteriori* tests; *p < 0.05.

In this study, statistically significant alterations of NMR spectrum peak areas were found for fatty acid groups, alanine, GABA, and glutamate during the progression of hMSC-derived chondrogenesis, as depicted in Figure 9. In the cases of leucine and phosphocholine, there were significant differences between the non-induced hMSCs and chondrogenic hMSCs older than 7 days. However, significant differences were not observed among the different chondrogenic hMSC groups. Therefore, these results do not seem to be sufficient to define leucine and phosphocholine as chondrogenic markers without comparisons of additional metabolomic changes.

Figure 9(a) shows the metabolomic variations of fatty acid 1 and fatty acid 2 during chondrogenic differentiation. One-way ANOVA tests for fatty acid 1 showed no significant changes in the non-induced hMSCs (control) or induced hMSCs at Day 4 or Day 7 (*p* > 0.05), whereas the metabolomic variation of fatty acid 1 increased after Day 7 (Figure 9[a], left; *p* < 0.05). For fatty acids 2–6, no significant alterations (*p* > 0.05) between the control and Day 4 groups were observed, although there was a significant increase after Day 4 (*p* < 0.05). Fatty acid 2 showed the highest intensity integral mean value and the most rapid increments among the fatty acids. All the fatty acids may represent a chondrogenic differentiation marker that allows the estimation of the second half of differentiation (after 4 days for fatty acids 2–6 and after 7 days for fatty acid 1), as their metabolomic variations increased with differentiation time. Statistical analyses of other metabolomic alterations along the chondrogenic process were also conducted. It was confirmed that significant metabolomic changes occurred for GABA, alanine, and glutamate, as shown in Figure 9(b). Alanine showed significant reductions in the control, Day 4, and Day 7 cells; there were no statistical variations after Day 7 (*p* > 0.05). GABA showed a trend towards increasing peak areas. GABA significantly increased during the first 4 days from the start of differentiation, as well as at the last two time-points, Day 11 and Day 15. During the intermediate time-points, from Day 4 to Day 11, no significant changes in peak area were found. In the case of glutamate, gradual differentiation was observed from Day 7 to Day 15, as evidenced by significant changes between Days 7, 11, and 15. Among the various metabolomes, the largest changes were seen for glutamate, with the exception of the fatty acids (Figure 8). Alanine showed clear differences at an early differentiation stage (before Day 7), whereas glutamate rapidly increased in a late differentiation stage (after Day 7). Therefore, alanine and glutamate may be complementary chondrogenic markers.

### Real-Time PCR

**Figure 10:**
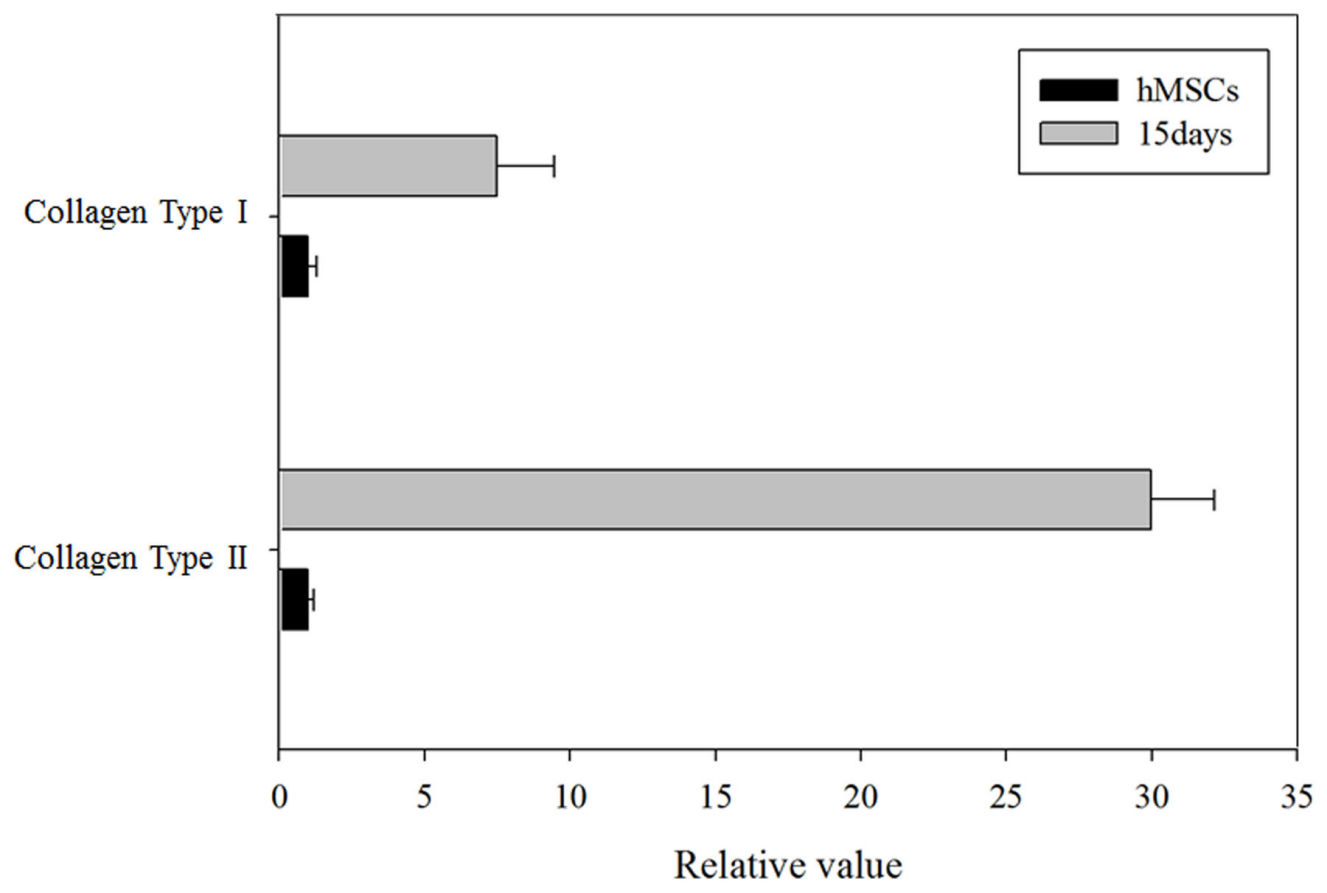
Real-Time PCR for the genes encoding collagen types I and II. The graph represents gene expression changes between non-induced hMSCs and hMSCs after 15 days of chondrogenesis. The level of hMSC gene expression was normalized to 1.0.

Real-Time PCR was performed to quantify the metabolomic changes in chondrogenic hMSCs. Figure 10 shows the gene expression levels for collagen types I and II. The expression level of each target gene was normalized to that of GAPDH. The gene expression level for each cell sample was normalized to that of non-induced hMSCs. The expression levels of the genes for collagen types I and II in chondrogenic hMSCs were much higher than those in non-induced hMSCs; indeed, the increased expression confirmed chondrogenic differentiation.

## Discussion

In the present study, quantitative measurements of cell metabolomes during chondrogenesis of hMSCs were performed using NMR spectroscopy. NMR markers of chondrogenesis that can be used to estimate the differentiation stages of stem cells (hMSCs) were identified. Previous reports have suggested that collagen expression increases with chondrogenesis progression [24,33]. Therefore, we hypothesized that the metabolomic changes indicated by NMR spectra would correspond to hMSC chondrogenesis. Although *in vivo* MRI has advantages, such as being noninvasive and allowing high-contrast imaging of soft tissue, its sensitivity levels at the cellular and molecular levels are low. Cell-based techniques that utilize a 3D scaffold mimicking physiologic conditions show promise [23]. It is well known that scaffolds play an important role in dictating cell adhesion, differentiation, and proliferation by interacting with cells and culture media. To overcome the obstacle of obtaining sufficient autologous chondrocytes and then expanding them *ex vivo* in a monolayer culture, and to stimulate the chondrogenic phenotype, a 3D culture system using biocompatible alginate scaffolds is commonly used, both *in vitro* and *in vivo*, due to its mild gelation mechanism, its structural similarity to the extracellular matrix, and its low toxicity when purified [22,27]. Recent work has demonstrated the encapsulation and differentiation of hMSCs, which turn into cartilage-forming chondrocytes [5]. In the present study, a 3D cell culture differentiation method was adopted to overcome the low sensitivity of clinical MRI machines owing to its high cell capacity per unit volume, as compared with 2D culture [34]. An NMR sensitivity test at 14.1 T using various cell densities presented a spectral peak that was clearly detectable at cell densities of greater than 2×10^6^ cells/mL, as shown in Figure 2. 

### Biological functions of metabolomes

NMR spectroscopy facilitates the observation of metabolomes at the molecular level. In the present study, we monitored metabolomic changes that occurred during chondrogenic differentiation of hMSCs using NMR spectroscopy. Various biological changes were observed at the molecular level, as evidenced by spectral peak changes. In general, during viscerogenic chemical reactions, fatty acids are used as a resource for the tricarboxylic acid cycle (TCA; also known as the Krebs or citric acid cycle). This allows the synthesis of ATP (adenosine triphosphate) by hydrolyzing triglycerides into fatty acids [35,36]. We deduced that ATP synthesis was associated with the increased fatty acid levels detected during the chondrogenic process.

Creatine is indicative of cellular storage and supplies energy to the cell, particularly in muscle and brain tissues. Its levels are increased by the decomposition of phosphocreatine when ATP is being synthesized for energy supply [37,38]. In the present study, the creatine peak decreased rapidly after chondrogenesis and was not detectable in chondrogenic hMSCs. We infer that high-energy metabolism, which uses creatine, is restrained in chondrogenic hMSCs. Phosphocholine, at 3.22 ppm, represents choline storage *in vivo* and is related to the size of the cell membrane. It is also involved in the synthesis of lecithin, which is a type of phosphatidylcholine, by reacting with CTP (choline-phosphate cytidylyltransferase) [39-41]. Lecithin is a fatty substance that makes up 30%–50% of all phospholipids. As shown in Figure 8, phosphocholine is increased during chondrogenic differentiation, although cell proliferation was not detected by either DNA assays or cell counting in the present study. Thus, the increased phosphocholine level during chondrogenesis was likely caused by hMSC differentiation into chondrocytes rather than by proliferation [42,43]. We infer that chondrocytes are bulkier than hMSCs, as the phosphocholine level relates to the size of the cell membrane, which covers the entire cell, and is higher in chondrocytes than in hMSCs that do not proliferate. Furthermore, this result suggests that changes in cell volume can be monitored through NMR observation. No phosphocholine signal was present in the alginate beads or media alone. This indicates that the phosphocholine originated only from the cells (in this case, hMSCs and chondrocytes). For this reason, it is expected that phosphocholine, which induces the synthesis of phospholipids associated with the cell membrane, can be used as an NMR proliferation marker without using standard materials, such as TSP.

### Chondrogenic differentiation marker

The levels of the following metabolomes were consistently altered: fatty acids, alanine, leucine, creatine, GABA, glutamate, and phosphocholine. In particular, fatty acids may represent an effective chondrogenic marker, as well as a marker that distinguishes between adipocytes and chondrocytes. A previous study showed that the metabolome contents of hMSCs changed after undergoing adipogenesis *in vitro*. Specifically, the level of fatty acid 1 increased 2.8-fold and 14-fold in two different studies, respectively. hMSCs undergoing chondrogenesis exhibited fewer metabolomic changes than those undergoing adipogenesis. The fatty acid levels increased during adipogenesis, although the peaks were distinct from those observed during chondrogenesis [20,21]. These results indicate that the differences in fatty acid profiles facilitate the discrimination of chondrogenesis and adipogenesis. 

The alanine and leucine levels gradually decreased over the entire time course. Although the alanine levels were significantly different compared with the non-induced hMSCs, the leucine levels were not, despite following a similar downward trend. However, both metabolomes retained relatively consistent levels compared with other (increased) metabolomes just after the beginning of differentiation. Thus, the initiation of chondrogenesis can be identified by a reduction in alanine levels. Leucine, which did not show a statistically significant change, could be a supplementary marker of chondrogenesis. The GABA and glutamate levels increased significantly compared with the alanine and leucine levels. There were significant differences in glutamate levels in the advanced-stage hMSCs. In the case of GABA, there was a significant difference between the non-induced hMSCs and chondrogenic hMSCs on Day 15, but not with cells at other time-points. Therefore, glutamate can be considered a chondrogenic marker that signals the final stage, in contrast to alanine. GABA may also be a chondrogenic marker of the later phase of chondrogenesis, as it showed significantly different levels in non-induced hMSCs versus chondrogenic hMSCs at Day 15.

## Conclusion

In conclusion, chondrogenic markers were identified for specific differentiation stages using NMR spectroscopic analysis of 3D-cultured hMSCs in alginate beads. We expect that the method used in this study will inform diagnostic techniques in the field of regenerative medicine, including cell therapy.
